# Feasibility and Safety of Endoscopic Vacuum Therapy of Walled-Off Necrosis in Acute Necrotizing Pancreatitis

**DOI:** 10.1055/a-2897-6337

**Published:** 2026-07-08

**Authors:** Peter Ewald, Oscar Cahyadi, Christoph Zeidler, Max Tophof, Mousa Ayoub, Markus W. Scheppach, Sandra Nagl, Jochen Wedemeyer, Diogo T. H. de Moura, Eduardo G. H. de Moura, Helmut Messmann, Waldemar Uhl, Alanna Ebigbo

**Affiliations:** 1 Department of Medicine and Gastroenterology 91789 Katholisches Klinikum Bochum Sankt Josef-HospitalBochumNordrhein-WestfalenGermany; 2 Department of Gastroenterology (III. Med. Klinik) 39694 University Hospital AugsburgAugsburgBayernGermany; 3 Department of Internal Medicine 39508 Robert-Koch-Hospital GehrdenGehrdenNiedersachsenGermany; 4 Gastrointestinal Endoscopy UnitHospital das Clínicas da Faculdade de Medicina da Universidade de São PauloSão PauloSão PauloBrazil; 5 Gastrointestinal Endoscopy Department 117265 Universidade de Sao Paulo Hospital das ClinicasSao PauloBrazil; 6 Department of Surgery 91789 Katholisches Klinikum Bochum Sankt Josef-HospitalBochumNordrhein-WestfalenGermany

**Keywords:** endoscopic ultrasonography, pancreas, intervention EUS, quality and logistical aspects, performance and complications

## Abstract

**Background** Endoscopic vacuum therapy (EVT) is a minimally invasive therapy for managing transmural gastrointestinal defects and supports the control of local infection. The role of EVT in patients with infected walled-off necrosis (WON) resulting from acute necrotizing pancreatitis (ANP) has not yet been investigated.

**Methods** A multicenter retrospective case analysis was conducted. Patients with infected WON due to severe ANP (Revised Atlanta Classification) in whom the standard step-up therapy was expanded to include EVT, were identified and included in the study.

**Results** Sixteen patients (aged 22–81 years) were treated in addition to standard therapy with EVT in four tertiary care centers in Germany and Brazil. After a total of 108 EVT device exchanges, the clinical success (sepsis control, complete resolution of WON, and closure of fistula) was achieved in 14 of 16 patients. Two patients died as a direct result of ANP and WON, while one further patient died of pneumosepsis after complete resolution of WON.

Intracavitary bleeding occurred during device exchange in two patients, corresponding to a per-procedure bleeding rate of 1.9%.

**Conclusions** Intracavitary EVT is feasible and relatively safe in treating patients with WON. Larger prospective trials are necessary to evaluate a potential adjunctive benefit of EVT in this challenging patient cohort.

## Introduction

Endoscopic vacuum therapy (EVT), also known as endoscopic negative-pressure therapy, represents a revolutionary minimally invasive approach for managing transmural gastrointestinal defects, including defects extending into the retroperitoneal space. 1 4 5 This innovative technique uses negative pressure wound therapy to achieve effective closure of complex gastrointestinal perforations while preserving organ function. 2 3 EVT has emerged as a viable alternative to traditional surgical management. 4 5

Infected walled-off necrosis (WON) represents one of the most challenging complications of acute necrotizing pancreatitis (ANP), with sepsis being a life-threatening consequence that requires prompt recognition and aggressive multidisciplinary management with mortality rates ranging from 15% to 40%, despite advances in intensive care management. 6 7 The high mortality is attributed to the development of multiple organ dysfunction syndrome, persistent systemic inflammatory response syndrome, and the technical challenges associated with source control in critically ill patients. 6 7 8

The management of sepsis caused by WON follows a systematic step-up approach that prioritizes minimally invasive interventions while maintaining the option for surgical escalation. This evidence-based strategy, validated by landmark trials, including the PANTER and TENSION trials, 9 10 has demonstrated superior outcomes compared to a primary open or minimally invasive surgical necrosectomy. EVT in the treatment of patients with infected WON and ANP has not been sufficiently addressed in the literature with only case reports available. 11 12 13 14 15 18 In this multicenter, retrospective study, we analyzed data from patients with WON and ANP who underwent EVT as an adjuvant treatment modality. The primary aim of the study was the clinical success of EVT, whereas the secondary aim was the intracavitary bleeding rate.

## Methods

This study was designed as a multicenter, retrospective case series. Data between 2022 and 2024 were collected from four tertiary care centers in Germany and Brazil. Patients with infected WON and acute pancreatitis (Revised Atlanta Classification), treated additionally with EVT, were included.

Clinical success was defined as sepsis control, complete resolution of WON, and closure of the fistula. Bleeding, a complication of WON, was defined as intracavitary bleeding requiring endoscopic, radiologic, or surgical treatment. Severe pancreatitis was defined according to the Revised Atlanta Classification, 16 and sepsis was defined according to the Sepsis-3 guidelines. 17

Data were obtained through the electronic patient reports and summarized using descriptive statistical parameters, including means for continuous variables and percentages for categorical variables. A positive ethics vote had been granted for retrospective case series by the Ethics Committee of the Ruhr-Universität Bochum.

## Endoscopic vacuum therapy

**Fig. 1:**
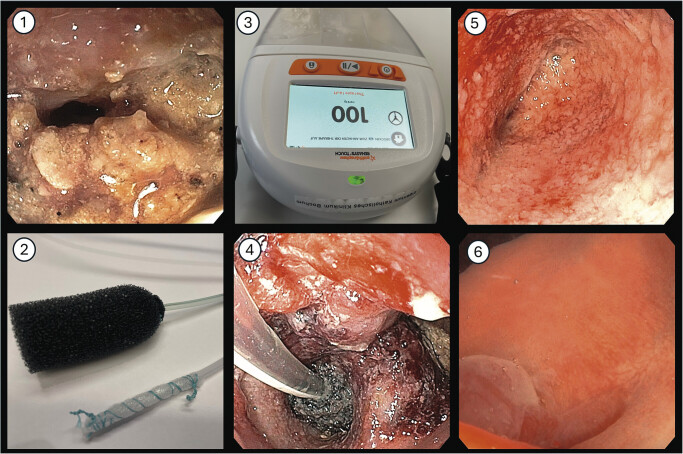
Stepwise endoscopic vacuum-assisted treatment of infected walled-off necrosis (WON) and colonic perforation: ( **1** ) debris within infected WON; ( **2** ) OPFD device and Endo-Sponge; ( **3** ) portable vacuum unit (RENASYS TOUCH, Smith & Nephew Ltd., UK); ( **4** ) Eso-Sponge debridement of WON cavity; ( **5** ) cleaned WON cavity; and ( **6** ) closed perforation of the left colon.

EVT was performed with a standard esophageal vacuum sponge (Eso-Sponge, B. Braun, Melsungen, Germany) in 10 patients. In six patients self-made open-pore film drainage (OPFD) were used. The choice of sponge system at each participating center was made by the investigators based on their personal experience with the respective device. The devices were placed into the WON or intraluminally adjacent to the WON at a negative pressure of −60 to −175 mmHg via a portable vacuum unit (Renasys Touch, Smith & Nephew LTD, UK ( Fig. 1 ). In one case, a vacuum stent (VacStent GI, Micro-Tech Europe) at a negative pressure of −80 mmHg was used. Access to the WON was achieved via an endoscopically placed lumen-apposing metal stent (LAMS), after spontaneous perforation of the WON into the gastrointestinal tract or following surgical cystogastrostomy. In one case, dilation of a percutaneous drainage tract enabled WON access. The LAMS was removed upon completion of EVT, at a time when the WON cavity was free of debris and had shrunk to only a few centimeters in size.

Sponge or OPFD exchange was done every 3–5 days until necrosis was no longer observed and/or sufficient size reduction of the WON was achieved. In case of WON perforation into the gastrointestinal tract, EVT was continued until 3–5 days after closure of the defect. In addition to EVT, direct endoscopic necrosectomy (DEN) was performed as indicated by the clinical course and according to the decision of the endoscopist, usually in parallel with sponge exchanges (in the interval between removal of the old device and placement of the new device), until the cavity was free of debris. In case of a VacStent GI, exchange was performed every 5–7 days. Regular imaging studies were done according to patients’ performance and clinical course of disease.

## Results

A total of 108 EVT sessions (exchange or removal of an EVT device) in 16 patients (10 male and 6 female patients) aged 22–81 years (mean age: 57.2 years) with infected WON and severe ANP were evaluated. ANP was attributable to alcohol in three patients, post-ERCP pancreatitis in three patients, cholelithiasis in three patients, and idiopathic or other causes in seven patients.

The decision to commence EVT in addition to standard endoscopic management was due to spontaneous perforation of WON into the gastrointestinal tract in five patients; life-threatening sepsis persisting despite maximal use of standard endoscopic therapy, in the absence of feasible surgical options, in five patients; extensive size of WON (diameter ≥ 15 cm) in four patients; and lack of a healing tendency under standard endoscopic therapy (treatment-refractory WON) in two patients. Patient characteristics are summarized in Table 1 .

**Table 1:** Summary of patient’s characteristics and therapy results.

No.	Age	Gender	Cause of pancreatitis: 1 = alcohol 2 = biliary 3 = idiopathic 4 = post-ERCP 5 = others	Indication for EVT: 1 = uncontrolled sepsis 2 = treatment refractory WON 3 = spontaneous perforation into stomach/intestinum 4 = large size WON	Size of WON in CT scan	Clinical success
1	56 y	Female	4	3 (perforation in left colon)	155.5 cm ^3^	Yes
2	64 y	Female	1	1	241.9 cm ^3^	Yes
3	22 y	Female	5 (Hyperlipidemia)	1	3041.1 cm ^3^	No (death day 37 of EVT-Therapy)
4	74 y	Male	3	1	245 cm ^3^	Yes
5	53 y	Female	4	1	471.2 cm ^3^	No (death day 15 of EVT-Therapy)
6	58 y	Male	5	3 (perforation in jejunum)	176.7 cm ^3^	Yes
7	74 y	Male	3	3 (perforation in stomach)	42.4 cm ^3^	Yes (death day 48 after WON resolution)
8	81 y	Male	3	4	275.9 cm ^3^	Yes
9	44 y	Male	1	4	1114 cm ^3^	Yes
10	56 y	Male	1	4	1361.3 cm ^3^	Yes
11	53 y	Female	2	3 (perforation in duodenum)	N/A	Yes
12	73 y	Female	2	4	241.9 cm ^3^	Yes
13	37 y	Male	5	2	199.8 cm ^3^	Yes
14	71 y	Male	2	2	2144.7 cm ^3^	Yes
15	42 y	Male	5 (following pancreatectomy)	1	523.6 cm ^3^	Yes
16	57 y	Male	4	3 (perforation in duodenum)	268.1 cm ^3^	Yes

EVT was initiated 17–119 days (mean 53.7 days, IQR 45.9 days) after hospitalization due to acute pancreatitis. Access to the WON cavity was obtained via transgastric/transduodenal LAMS in seven cases, via WON perforation in five patients (1× stomach, 2× duodenum, 1× jejunum and 1× colon), and following surgical cystogastrostomy in four patients. No additional balloon dilatation of the LAMS was required at the start of EVT, because standard endoscopic therapy had been performed in all patients for several weeks.

The size of the WON measured in the CT scan was 42.4–3041.1 cm ^3^ (mean 700.2 cm ^3^ ).

The primary outcome of clinical success (sepsis control, complete resolution of WON, and closure of the fistula) was achieved in 14 of 16 patients within a median therapy duration of 31.7 days (range: 3–101 days). Two patients died on days 15 and 37, respectively, after EVT onset due to ongoing and uncontrollable sepsis. One additional patient who had been successfully treated per protocol died 2 months after complete resolution of his paragastric WON due to pulmonary sepsis.

The mean intensive care unit stay was 70.7 days (range: 3–162 days). During the period of EVT, between 1 and 26 EVT sessions were performed per patient, resulting in the total use of 87 Eso-Sponge, 38 individual OPFD devices, and four VacStent GI. All OPFD devices were placed into the WON and all VacStent GI were placed intraluminally and adjacent to the entrance into the WON. Seventy-eight Eso-Sponge devices were placed into the WON and nine into the lumen adjacent to the WON. The four VacStents GI devices were used in a patient with an infected WON, in whom coils from a previously performed endovascular embolization of a splenic arterial aneurysm became visible after intracavitary Eso-Sponge therapy. In fact, Eso-Sponge devices were exchanged on average every 4.2 days, OPFD devices on average every 5.3 days.

In addition, 12 of 16 patients underwent at least one session of DEN, with a total of 81 necrosectomy procedures performed (mean 6.8 DEN per patient). Details of the procedures are summarized in Table 2 .

**Table 2:** Characteristics of EVT therapy.

No.	EVT device 1 = Eso-Sponge 2 = VacStent GI 3 = OPFD-Device	Position of EVT device 1 = intracavitary 2 = intraluminal/paracavitary	Number of sponges/exchanges	Duration EVT	Negative pressure settings (mmHg)	EVT application route 1 = transgastral 2 = transduodenal 3 = transcutaneous 4 = others	Bleeding	DEN	Local results 1 = WON resolution 2 = no improvement
1	1 (42×)	1 (41×), 2 (1×)	42/25	101d	80	4 (transcolonic)	None	19	1
2	1 (3×)	1 (3×)	3/2	14d	60	1	None	5	1
3	1 (9×)	1 (9×)	9/8	34d	120	1 and 3	Yes	12	2
4	1 (8×)	1 (8×)	8/7	31d	80	1	None	6	1
5	1 (2×)	1 (2×)	2/1	12d	60	2	None	–	2
6	1 (11×), 2(4×)	1 (4×), 2 (11×)	15/9	57d	80	4 (transjejnunal)	None	2	1
7	1 (2×)	1 (2×)	2/1	7d	80	1	None	–	1
8	1 (3×)	1 (3×)	3/2	11d	60	1	None	2	1
9	1 (1×)	1 (1×)	1/0	3d	80	1	None	2	1
10	1 (5×)	1 (5×)	5/4	25d	80	1	None	3	1
11	3 (3×), 1(1×)	1 (3×), 2(1×)	4/3	28d	70–125	2	None	–	1
12	3 (2×)	1 (2×)	2/1	14d	60	1	Yes	12	1
13	3 (6×)	1 (6×)	6/5	19d	125	2	None	7	1
14	3 (16×)	1 (16×)	16/15	80d	175	1	None	8	1
15	3 (6×)	1 (6×)	6/5	32d	175	1	None	3	1
16	3 (5×)	1 (5×)	5/4	28d	175	2	None	–	1

In 108 EVT sessions (device implantation and exchange) and 81 necrosectomies, relevant intracavitary bleeding occurred in two patients. In one patient, following Eso-Sponge device removal, bleeding was successfully managed endoscopically using a standard hemoclip. In the second patient, bleeding occurred following OPFD device removal. Endoscopic treatment was insufficient and bleeding was finally treated with interventional angiography.

## Discussion

In this retrospective case series of patients with sepsis due to infected WON and ANP, we aimed to assess the feasibility and clinical outcomes of adjunctive EVT in addition to standard therapy. EVT has emerged as a viable alternative to traditional surgical management for leaks in various locations of the upper and lower gastrointestinal tract 1 4 5 ; however, its potential in WON and ANP has not been sufficiently addressed.

Severe acute pancreatitis is a critical illness associated with high morbidity and mortality rates. After resolution of the early complications of acute pancreatitis, infection of pancreatic fluid and necrotic collections can lead to life-threatening situations. WON infection with sepsis is a serious clinical condition with mortality rates ranging from 15% to 40%, despite advances in intensive care management. 6 7 8

In addition to aggressive resuscitation and broad-spectrum antibiotic therapy, the core of treatment is the drainage and evacuation of the septic focus. Management usually follows a systematic step-up approach, prioritizing minimally invasive interventions while maintaining the option for surgical escalation. This evidence-based strategy was validated by landmark trials such as the PANTER and TENSION trial. 9 10

The use of EVT in the duodenum has been occasionally reported in a small series of patients with acute leaks resulting from perforated ulcers, iatrogenic perforations after endoscopic resection, or complications following upper gastrointestinal surgery. 1 4 5 However, there are only a few case reports describing the use of intraluminal EVT directly in the retroperitoneal space in the setting of infected WON. 13 14 15 18

Between 2022 and 2024, we performed retroperitoneal intracavitary vacuum therapy in 16 patients with infected WON. We expanded the standard therapeutic protocol by incorporating EVT in cases where there was a suitable access route from the gastrointestinal tract to the WON cavity, sufficiently large to allow the passage and uncomplicated removal of the Eso-Sponge or the OPFD device. Additionally, the patient needed to tolerate the inherent procedural discomfort of EVT, such as a transnasal tube and limited mobility due to the vacuum pump.

Medical indications for EVT included perforations of WON into the gastrointestinal tract, patients with extensive WON (diameter ≥ 15 cm), and patients with sepsis refractory to standard endoscopic therapy and the absence of feasible surgical options. All cases were discussed with abdominal surgeons at a multidisciplinary board.

In our study, multimodal treatment with adjunctive EVT led to clinical success (including sepsis control, complete resolution of WON, and closure of the fistula) in 14 of 16 patients within a mean treatment duration of 31.7 days. Unfortunately, two patients died during continuing EVT at 15 and 37 days after initiation, respectively, due to ongoing and uncontrollable sepsis.

One additional patient who had been successfully treated per protocol died 2 months after complete resolution of paragastric WON due to pulmonary sepsis. The observed overall mortality rate of 18.8% in our series is consistent with the literature, where mortality rates of 15–40% are described for infected WON complicated by severe sepsis 6 7 and 28% in patients with infected necrosis due to necrotizing pancreatitis. 19 Mortality increases in cases of organ failure and in patients undergoing open necrosectomy. 19 20

In the management of pancreatic fluid collections and WON, EVT was previously recommended as a rescue modality due to the risk of hemorrhage associated with proximity to major retroperitoneal vessels. 2 In our cohort, among 108 EVT sessions (device implantation and exchange) and 81 DEN, clinically significant intracavitary bleeding occurred in only two patients. Bleeding was successfully controlled in both cases. Although the per-patient bleeding rate was 12.5%, the bleeding rate relative to the number of EVT interventions (per-procedure bleeding rate) was only 1.9%.

In comparison, a prospective study including 52 patients with upper gastrointestinal defects treated with EVT (390 sponge insertions) reported a risk of bleeding per procedure of 1.8% (five minor and two major bleedings resulting in patient death). 21 The risk of clinically significant bleeding in our study using intracavitary EVT in WON is comparable to that reported for the well-established application of EVT in the treatment of upper gastrointestinal defects and appears to be lower than anticipated.

Although our data are promising, the small sample size of 16 patients, the retrospective analysis of a heterogeneous cohort and a lack of a control group make it difficult to determine the independent distribution of EVT to this therapeutic success. Moreover, EVT “accelerates” the endoscopic standard therapy, because DEN was performed in parallel in the interval of sponge exchanges (Eso-Sponge devices on average every 4.2 days, OPFD devices on average every 5.3 days). Current data from a randomized, controlled trial have demonstrated the benefits of accelerated clearance of infected WON. 22 New technical developments, such as large-sized LAMS and OPFD devices with a smaller diameter 1 may facilitate the technical outcome of EVT in WON.

During data collection, a total of 12 tertiary and secondary care centers in Germany, the Netherlands, Denmark, and Brazil were approached for possible patient enrollment. Although all centers agreed on the high clinical relevance of the study aims, only four centers had implemented EVT in patients with WON and ANP.

The data from this study, which demonstrate the feasibility and relative safety of EVT in the management of WON, should encourage further investigation within a prospective controlled study setting to assess the independent beneficial contribution of EVT as an adjunctive option in addition to standard endoscopic management.
